# The lateral distance between a proton pump and ATP synthase determines the ATP-synthesis rate

**DOI:** 10.1038/s41598-017-02836-4

**Published:** 2017-06-07

**Authors:** Johannes Sjöholm, Jan Bergstrand, Tobias Nilsson, Radek Šachl, Christoph von Ballmoos, Jerker Widengren, Peter Brzezinski

**Affiliations:** 1 Department of Biochemistry and Biophysics, The Arrhenius Laboratories for Natural Sciences, Stockholm University, SE-106 91 Stockholm, Sweden; 2 Experimental Biomolecular Physics, Department of Applied Physics, Royal Institute of Technology (KTH), SE-106 91 Stockholm, Sweden; 3 Department of Biophysical Chemistry, J. Heyrovský Institute of Physical Chemistry of the A.S.C.R. v.v.i., Prague, Czech Republic; 4 Department of Chemistry and Biochemistry, University of Bern, 3012 Bern, Switzerland

## Abstract

We have investigated the effect of lipid composition on interactions between cytochrome *bo*_3_ and ATP-synthase, and the ATP-synthesis activity driven by proton pumping. The two proteins were labeled by fluorescent probes and co-reconstituted in large (*d* ≅ 100 nm) or giant (*d* ≅ 10 µm) unilamellar lipid vesicles. Interactions were investigated using fluorescence correlation/cross-correlation spectroscopy and the activity was determined by measuring ATP production, driven by electron-proton transfer, as a function of time. We found that conditions that promoted direct interactions between the two proteins in the membrane (higher fraction DOPC lipids or labeling by hydrophobic molecules) correlated with an increased activity. These data indicate that the ATP-synthesis rate increases with decreasing distance between cytochrome *bo*_3_ and the ATP-synthase, and involves proton transfer along the membrane surface. The maximum distance for lateral proton transfer along the surface was found to be ~80 nm.

## Introduction

Oxidative breakdown of nutrients is the major supply for energy in living organisms. An intermediate in this energy conversion process in biological systems is a transmembrane proton electrochemical gradient that is composed of a proton-concentration gradient and an electrical membrane potential. This gradient is maintained by membrane-bound proton pumps or transporters and it is consumed by the F_1_F_o_ ATP-synthase (consumer), which converts ADP into ATP (for review, see ref. 1). The gradient is also used for other energy-requiring processes such as transmembrane transport or signaling. *A priori*, the chemiosmotic theory does not require any other role for the membrane than to comprise a proton-tight barrier. Likewise, the distance between the proton transporter/pump and the proton consumer is irrelevant when considering the overall energy-conservation process as long as both membrane proteins reside in the same membrane surrounding a closed compartment. However, there may be a kinetic advantage if protons would be transferred directly from the proton transporter/pump to the consumer faster than equilibrating with the bulk water solvent. A direct transfer mechanism requires proton transfer along the membrane surface to be significantly faster than the equilibration of protons with bulk solution. Briefly, parameters that are relevant to consider in this context is the composition of the membrane head groups, the water layer adjacent to the membrane surface, the average distance between proton transporters and consumers and the proton diffusion coefficient (reviewed in refs 2–4).

Surface proton conduction was observed in an early study with purple membranes of *H*. *salinarium*, where protons released upon illumination of the light-driven pump bacteriorhodopsin were transferred faster along the membrane surface than equilibrating with bulk water^5^. After the initial experimental observation, the phenomenon has been investigated in detail, both experimentally and theoretically^2, 3, 6–25^.

**Figure 1:**
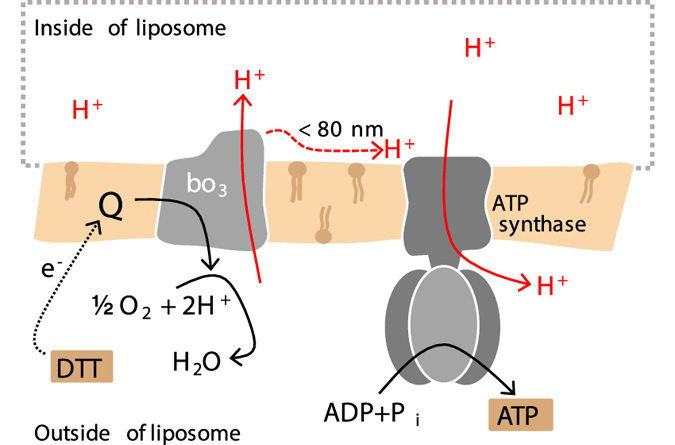
The experimental system. Cyt. *bo*_3_ and ATP-synthase from *E*. *coli* were co-reconstituted in vesicles (a part of the membrane is shown). For measurements of protein-protein interactions, cyt. *bo*_3_ and ATP-synthase were labeled with fluorophores (not shwon, see text). To measure the coupled activity, DTT and quinone were added, which initiates transmembrane proton transfer, driven by the quinol oxidase. The ATP-synthesis rate was monitored by measuring changes in luminescence that originates from added luciferase/luciferin. Proton transfer along the membrane surface is discussed in the Discussion section.

We have recently investigated proton-coupled ATP synthesis in a minimal model of a respiratory unit that is able to produce ATP from supplied electrons via redox-driven proton pumping^26, 27^ (Fig. 1). In this model system, cytochrome (cyt.) *bo*_3_ (ubiquinol oxidase) and F_1_F_o_ ATP synthase, both purified from *E*. *coli*, were co-reconstituted in vesicles of different lipid composition. The data showed that the rate of ATP-production decreased by a factor of ~10 upon addition of e.g. 1,2-dioleoyl-sn-glycero-3-phospho-(1′-rac-glycerol) (DOPG) to vesicles composed of only 1,2-dioleoyl-sn-glycero-3-phosphocholine (DOPC). Because the overall thermodynamic conditions were the same with the different lipids, the decrease in ATP production was explained in terms of a kinetic effect, indicating that protons were transferred along the membrane surface. However, the data from these earlier studies could not explain the observed effect at a molecular level. In the present study we investigated a possible link between the lipid head group composition, the average protein-protein distance and ATP-synthesis activity using fluorescence correlation spectroscopy (FCS) and fluorescence cross-correlation spectroscopy (FCCS). We labeled cyt. *bo*_3_ and ATP-synthase with fluorescent probes that were used both to detect their interactions in an essentially planar unilamellar membrane, and also to promote protein-protein interactions via contacts between the hydrophobic probes. Changes in average distance were correlated with changes in average activity. The data indicate that the average distance between the proton pump and ATP-synthase depends on lipid composition, and the ATP-synthesis rate increases with decreasing distance between cyt. *bo*_3_ and ATP-synthase. The maximum average distance for proton diffusion at the membrane surface was ~80 nm.

## Results

### Protein reconstitution in giant unilamellar vesicles

To investigate protein-protein interactions in lipid membranes, cyt. *bo*_3_ and ATP-synthase were co-reconstituted in giant unilamellar lipid vesicles (GUVs) with a diameter of ~10 μm, using a modified protocol of Dezi *et al*.^28^ (for review, see ref. 29). Here, the GUVs were formed in a sucrose solution from dried lipid films placed on gold-covered glass plates by applying a voltage across the liquid (usually referred to as electroformation). The proteins, solubilized in detergent, were incorporated by dilution of the detergent (described in detail in the “Materials and Methods” section). The diameter of these vesicles is such that the membrane surface is essentially planar in the measuring area of the confocal FCS setup. The microscope set-up allows for detection of two fluorophores at a time, and thus a cross correlation analysis of cyt. *bo*_3_ (labeled with either ATTO 647N or Abberior STAR 635) and ATP synthase (labeled with ATTO 594), i.e. both diffusion and co-diffusion of the protein complexes could be studied.

**Figure 2:**
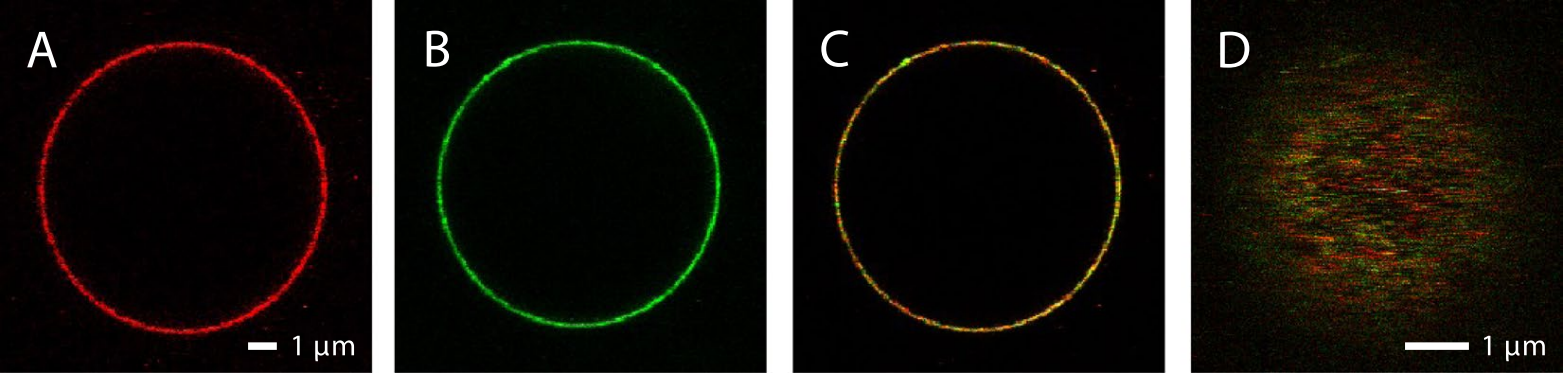
Confocal scanning microscope images of a GUV in which two fluorophore-labeled proteins were reconstituted. (**A**) Detection of cyt. *bo*_3_ labeled with ATTO 647N. The focal plane is at the middle of the vesicle. (**B**) Detection of ATP-synthase labeled with ATTO 594. (**C**) Combined image of ATTO 594 and ATTO 647N detection. (**D**) An image of the top of the vesicle, which is the focal plane used for the FCS measurements. The lipid composition of the vesicle was 99% DOPC and 1% DPPE functionalized with a biotinyl head group. The GUVs were immobilized on a streptavidin-coated cover slide and the solution around the vesicle was 10 mM HEPES pH 7.4, supplemented with 10 mM NaCl and 100 mM glucose.

Figure 2 shows confocal laser scanning microscope images of a GUV with co-reconstituted cyt. *bo*_3_ labeled with ATTO 647N (panel A, red) and ATP synthase labeled with ATTO 594 (panel B, green). In panel C (yellow), an overlay of panels A and B is shown. Using a water-immersion objective, the focal plane could be put in solution on top of the vesicle (Fig. 2D). In this essentially planar section of the membrane we measured fluorescence-intensity fluctuations in different lipid environments.

### Protein diffusion in the lipid bilayer

**Figure 3:**
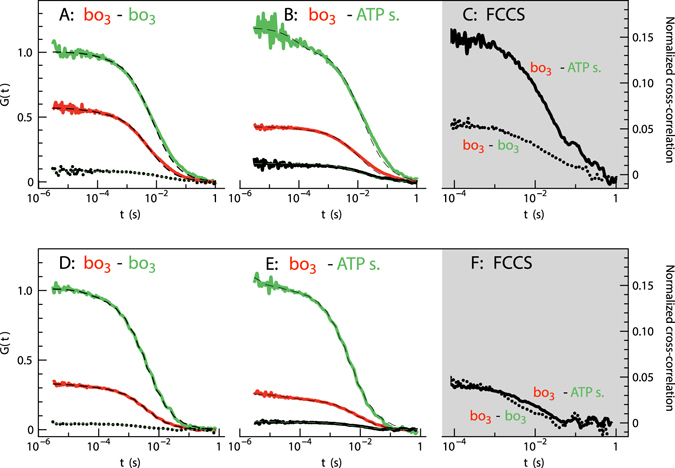
Auto-correlation data measured with GUVs containing co-reconstituted cyt. *bo*_3_ and ATP synthase. GUVs were composed of either 99% DOPC (**A**–**C**) or 94% DOPC and 5% DOPG (**D**–**F**), with the addition of 1% DPPE functionalized with a biotinyl head group. Measurements were done at pH 7.4 in 10 mM HEPES supplemented with 10 mM NaCl and 100 mM glucose. (**A**,**D**) FCS was used to study samples where cyt. *bo*_3_ was labeled with either ATTO 647N (red trace) or ATTO 594 (green trace). (**B**,**E**) samples with cyt. *bo*_3_ labeled with ATTO 647N and ATP-synthase labeled with ATTO 594. The dashed lines represent best fits of the data using a single component with planar two-dimensional diffusion and a triplet state fraction. The amplitude of the diffusional component obtained from the fit of the data with the ATTO 594-labeled protein has been set to unity (amplitude at ~10^−4^ s) to facilitate comparison of the traces. The autocorrelation function for FCCS was calculated in all cases and the normalized cross correlation amplitudes are compared in panels (**C**) and (**F**).

The diffusion of the proteins in the membrane was measured using FCS in two sets of experiments. In one experiment, only cyt. *bo*_3_ was present in the vesicles, but half of the population was labeled with ATTO 647N and the other half with ATTO 594. In the other experiment, cyt. *bo*_3_, labeled with either ATTO 647N or Abberior STAR 635, and ATP synthase, labeled with ATTO 594, were co-reconstituted in the GUVs. The stoichiometry of the proteins was kept constant between the different experiments, with a 2–3-fold excess of cyt. *bo*_3_ compared to the ATP synthase. In cases where only cyt. *bo*_3_ was present in the membrane the stoichiometry of the two fluorescent dyes was kept approximately the same, i.e. a 2–3 fold excess of ATTO 647N or Abberior STAR 635 compared to ATTO 594. Auto-correlation curves G(τ), calculated from the detected fluorescence intensity time traces (see “Materials and Methods” and ref. 30) are shown for the co-reconstituted proteins in DOPC vesicles (Fig. 3A,B) or DOPC:DOPG(5%) vesicles (Fig. 3D,E). These curves were fitted with a two-dimensional diffusion model in order to estimate the average number of particles in the detection area (*N*) and the average diffusion time of the fluorescent molecules through this area, τ_D_:

1 $${\rm{G}}({\rm{\tau }})=\frac{1}{{\rm{N}}(1-{\rm{T}})}{[1+\frac{{\rm{\tau }}}{{{\rm{\tau }}}_{{\rm{D}}}}]}^{-1}[1-{\rm{T}}+{{\rm{Te}}}^{-\frac{{\rm{\tau }}}{{{\rm{\tau }}}_{{\rm{T}}}}}]$$

It is assumed that the fluorophores exhibit a “dark”, non-fluorescent triplet state of fraction *T* with a relaxation time, τ_T_. The diffusion coefficient *D* was calculated from the diffusion time τ_D_, using the known lateral 1/e^2^ radius of the detection area (*w*):

2 $${{\rm{\tau }}}_{{\rm{D}}}=\frac{{{\rm{w}}}^{2}}{4{\rm{D}}}$$

**Figure 4:**
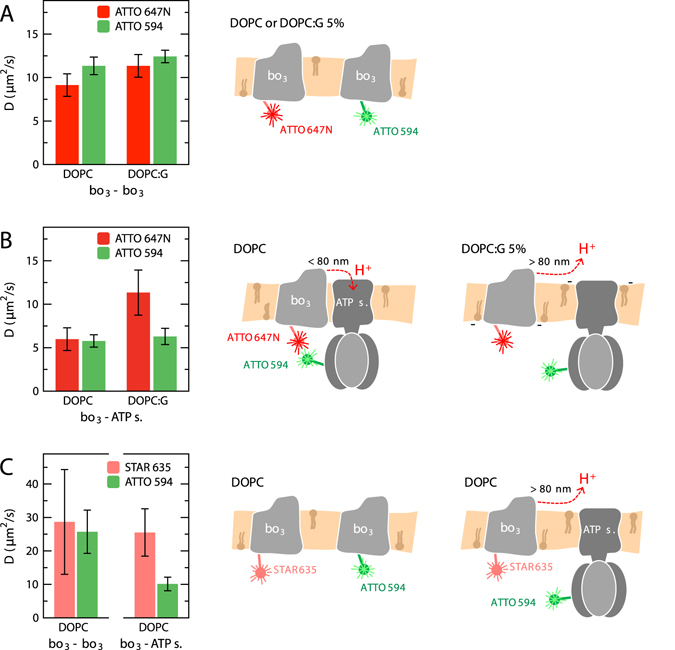
Diffusion coefficients determined from measurements of lateral diffusion of co-reconstituted proteins. (**A**) GUVs containing two populations of cyt. *bo*_3_, each labeled with either ATTO 647N or ATTO 594. The lipid composition was 99% DOPC or 94% DOPC and 5% DOPG (DOPC:G), and in addition 1% DPPE functionalized with a biotinyl head group. (**B**) Cyt. *bo*_3_ labeled with ATTO 647N and ATP-synthase labeled with ATTO 594. (**C**) Left: GUVs composed of DOPC with two populations of cyt. *bo*_3_ labeled with either Abberior STAR 635 or ATTO 594 (*bo*_3_-*bo*_3_). Right: GUVs composed of DOPC with cyt. *bo*_3_ labeled with Abberior STAR 635 and ATP-synthase labeled with ATTO 594 (*bo*_3_-ATP s.). Schemes to the right illustrate the conditions of the experiments and the conclusions (see text for details); ATTO 647N promotes the binding between cyt. *bo*_3_ and ATP synthase in DOPC membranes but not in DOPC:G membranes, reflected in the apparent diffusion constants of cyt. *bo*_3_ (**B**). The effect is not observed with only cyt. *bo*_3_ in the membrane (**A**) or when ATTO 647N is replaced by the more hydrophilic Abberior STAR 635 (**C**). Measurements were done at pH 7.4 in 10 mM HEPES supplemented with 10 mM NaCl and 100 mM glucose. Error bars represent standard deviation from measurements with five samples.

The measured diffusion coefficients of cyt. *bo*_3_ and ATP-synthase, labeled with two different ATTO dyes are shown in Fig. 4 (the dyes are indicated on the right-hand side schemes). The following observations were made:

(i) The diffusion coefficient of cyt. *bo*_3_ was, within experimental error, independent on the labeling dye (ATTO 647 N or ATTO 594), and similar in both DOPC and DOPC:DOPG(5%) membranes (Fig. 4A).
(ii) The diffusion coefficient of cyt. *bo*_3_ was a factor of two lower upon co-reconstitution of cyt. *bo*_3_ with the ATP-synthase in a DOPC membrane (Fig. 4B, two left-hand side bars, cyt. *bo*_3_ diffusion is slowed to the same value as that of the ATP-synthase).
(iii) The diffusion coefficient of cyt. *bo*_3_ was unaltered upon co-reconstitution with ATP synthase when 5% DOPG was present in the membrane (compare the red bars in panels A and B in Fig. 4 for DOPC:DOPG(5%)), while the diffusion coefficient of the ATP synthase remained the same as in the GUVs prepared from only DOPC.
(iv) When cyt. *bo*_3_ was labeled with the dye Abberior STAR 635 (more hydrophilic than the dyes used in the measurements described in points (i)–(iii) above), the diffusion coefficient of cyt. *bo*_3_ was unaltered upon introduction of the ATP synthase (Fig. 4C), also when measured in a membrane composed of only DOPC (compare to the data in Fig. 4B).

The schemes on the right-hand side of Fig. 4 summarize these observations: cyt. *bo*_3_ and ATP-synthase interact within the membrane, but only when labeled with the hydrophobic^31, 32^ ATTO 647N dye in pure DOPC. When using the more hydrophilic dye Abberior STAR 635 to label cyt. *bo*_3_ the interactions were lost. Similarly, the cyt. *bo*_3_-ATP-synthase interactions were lost upon addition of DOPG when cyt. *bo*_3_ was labeled with the hydrophobic dye ATTO 647N.

### Correlation of protein diffusion

**Figure 5:**
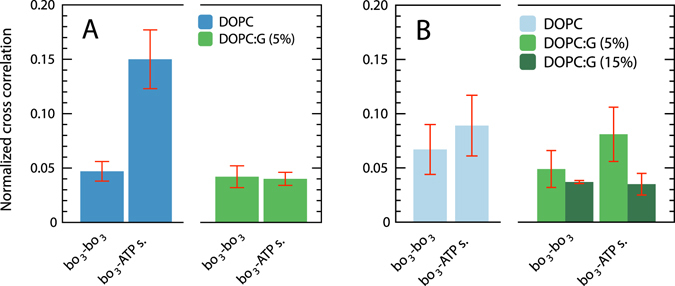
Summary of normalized cross correlation amplitudes measured with GUVs with co-reconstituted cyt. *bo*_3_ and ATP synthase. (**A**) The normalized amplitudes were calculated from samples with cyt. *bo*_3_ labeled with either ATTO 647 N or ATTO 594 (*bo*_3_-*bo*_3_), and cyt. *bo*_3_ labeled with ATTO 647N and ATP-synthase labeled with ATTO 594 (*bo*_3_-ATP s.). (**B**) Cyt. *bo*_3_ was labeled with Abberior STAR 635. The lipid composition was either 99% DOPC (DOPC, blue) or 84–94% DOPC and 5–15% DOPG (DOPC:G, green), and in addition 1% DPPE functionalized with a biotinyl head group. Measurements were done at pH 7.4 in 10 mM HEPES supplemented with 10 mM NaCl and 100 mM glucose. Error bars represent standard deviation from measurements with five samples.

To investigate whether or not the diffusion of cyt. *bo*_3_ and ATP-synthase were correlated, we measured the cross-correlation of the fluorescence intensity fluctuations from two different dyes attached to cyt. *bo*_3_ and ATP-synthase, respectively, using FCCS (measured simultaneously with FCS). In Fig. 3C and F, the normalized (see “Materials and Methods” section, Equation 6) cross-correlation amplitudes are shown. We compare GUVs with only cyt. *bo*_3_ (dotted lines, two populations of cyt. *bo*_3_, each labeled with either ATTO 647N or ATTO 594) to those with both cyt. *bo*_3_ and ATP-synthase (solid lines, cyt. *bo*_3_ and ATP-synthase labeled with ATTO 647N and ATTO 594, respectively) in membranes composed of only DOPC (panel C in Fig. 3) or DOPC: DOPG(5%) (panel F). In pure DOPC the normalized cross-correlation amplitude measured with membranes containing only cyt. *bo*_3_ was found to be a factor of ~3 lower than that measured in membranes containing both cyt. *bo*_3_ and ATP-synthase (Fig. 3C). No such amplitude differences could be observed upon introduction of 5% DOPG into the membrane (Fig. 3F). Furthermore with 5% DOPG, the normalized cross-correlation amplitude for the cyt. *bo*_3_ and ATP-synthase was about the same as that for cyt. *bo*_3_ only (two populations of cyt. *bo*_3_, each labeled with a different dye, summarized in Fig. 5A). Furthermore, the data in Fig. 5B shows that only a moderate increase in the normalized cross-correlation amplitudes could be noted upon addition of ATP-synthase (labeled with ATTO 594) to membranes containing cyt. *bo*_3_, labeled with the more hydrophilic Abberior STAR 635 dye. The amplitudes were slightly lowered upon addition of 5% or 15% DOPG to the DOPC membranes.

### Coupled enzymatic activity

**Figure 6:**
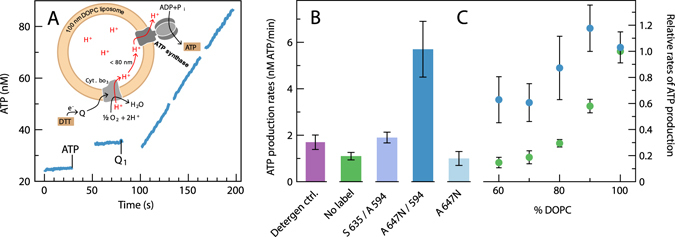
Coupled cyt. *bo*_3_-ATP synthase activity. (**A**) ATP production by the ATP synthase, driven by an electrochemical gradient generated by cyt *bo*_3_ (as shown in the scheme). ATP synthesis was measured as a change in luminescence from the luciferin-luciferase couple over 3 × 30 s. The reactions were started by the addition of ubiquinol Q_1_H_2_ (20 μM final concentration) in the presence of 2 mM DTT and 80 μM ADP. Measurements were done at pH 7.5 in 20 mM Tris-PO_4_ buffer supplemented with 2.5 mM MgCl_2_. Rates were calculated from the average slopes, calibrated by addition of well-defined amount of ATP (5 pmol, see mark at 30 s). The trace shown was obtained with 100 nm, 100% DOPC liposomes. (**B**) ATP-synthesis rates measured in DOPC vesicles with cyt. *bo*_3_ labeled with either ATTO (A) 647N (and unlabeled ATP synthase) or Abberior STAR 635 (and ATP-synthase labeled with ATTO 594). Rates are compared to those obtained with proteoliposomes with unlabeled cyt. *bo*_3_ and ATP-synthase in the presence (2.5 μM DDM, Detergent ctrl., see text for explanation) or absence (No label) of detergent. (**C**) Normalized ATP-synthesis rates of DOPC:DOPG vesicles with cyt. *bo*_3_ and ATP-synthase labeled with ATTO 647N and ATTO 594 respectively (blue) or vesicles with unlabeled protein (green). The rates are normalized to that obtained with 100% DOPC vesicles to facilitate comparison. At 100% DOPC the activity was a factor of ~5 larger with the labeled than with the unlabeled proteins (c.f. panel B). Error bars is the standard deviation from measurements with four samples (except Detergent ctrl., two samples).

To investigate a possible link between the average protein-protein distance and activity, we investigated the rates of ATP-synthesis, driven by proton pumping by cyt. *bo*_3_ (referred to as the “coupled activity”), under similar conditions to those used in the studies described above. The fluorophore-labeled cyt. *bo*_3_ and ATP synthase where co-reconstituted in large (diameter ~100 nm) unilamellar vesicles. The protein concentration was adjusted to approximately five proteins of each per vesicle, i.e. the same as that used in our earlier studies (see refs 26 and 27). The coupled cyt. *bo*_3_-ATP synthase activity was measured by monitoring the ATP-production rate upon addition of ubiquinol Q_1_/DTT, which reduces cyt. *bo*_3_, leading to transmembrane charge separation and proton pumping in the presence of O_2_ (Fig. 6A). It should be noted that the dyes were used in these specific measurements only to investigate the effect of introducing hydrophobic probes that promote direct protein-protein interactions (and use the same conditions as those in the FCS-measurements discussed above).

As seen in Fig. 6B, when ATTO 594 labeled ATP-synthase and ATTO 647N labeled cyt. *bo*_3_ were co-reconstituted, the coupled activity was about a factor of five higher than that measured with unlabeled proteins (c.f. “control” in Fig. 6B). This increase in coupled activity was much smaller (less than a factor of two) when cyt. *bo*_3_ was instead labeled with the more hydrophilic dye Abberior STAR 635 (ATP-synthase was still labeled with ATTO 594).

Next, we studied the coupled activity as a function of the lipid composition, i.e. with increasing amounts of DOPG, (0-40%, Fig. 6C) added to vesicles composed of DOPC. As observed previously^26, 27^, there was a clear decrease in the ATP-synthesis rate with increasing concentrations of DOPG. However, upon labeling cyt. *bo*_3_ with ATTO 647N and the ATP-synthase with ATTO 594, the dependence on lipid composition was significantly less pronounced. Upon decreasing the fraction of DOPC from 100% to 60%, the ATP-synthesis rate dropped to ~60% for the labeled proteins, as compared to ~10% for the unlabeled proteins (Fig. 6C).

In the experiments discussed here, the detergent was removed by gel chromatography during vesicle reconstitution, while during reconstitution in GUVs the detergent concentration was decreased below CMC by dilution. To investigate whether or not the presence of remaining small amounts of detergent (~2.5 μM DDM) altered the coupled enzymatic activity, the ATP production was measured also in the presence of the detergent DDM at the same low concentration (Fig. 6B, “Detergent ctrl.”). As seen in the Figure, the coupled activity was about the same with and without detergent.

A few relevant controls should be mentioned. Binding of the dyes did not alter the activities of the two enzymes alone. The activity of cyt. *bo*_3_ was measured using a Clark-type electrode and was found to be about the same with (460 ± 50 e^−^/s) and without (440 ± 60 e^−^/s) the label ATTO 647 N. The data in Fig. 6B show that the coupled cyt. *bo*_3_-ATP-synthase activity was about the same for unlabeled proteins as upon labeling cyt. *bo*_3_ with Abberior STAR 635 and ATP-synthase with ATTO 594, i.e. labeling of these proteins with the non-interacting dyes did not alter the activity indicating that the activity of the ATP-synthase was unaltered upon labeling with ATTO 594. Furthermore, the coupled activity was essentially unaltered upon labeling cyt. *bo*_3_ with ATTO647N leaving the ATP-synthase unlabeled. Taken together, these data indicate that neither labeling of cyt. *bo*_3_ nor of ATP-synthase did result in altering the activity of these enzymes. Furthermore, because the coupled cyt. *bo*_3_-ATP-synthase activity was insensitive to labeling of one of the proteins at a time or with non-interacting dyes, the data suggest that neither the relative orientation nor proton leaks were influenced by the labeling.

## Discussion

As outlined in the Introduction section, in a recent study we found that the coupled enzymatic activity of cyt. *bo*_3_ and ATP synthase (Fig. 1) was dependent on the lipid composition of the vesicles; the activity dropped by a factor of ~10 upon addition of e.g. 40% DOPG to DOPC vesicles with a diameter of 100 nm. On the basis of these findings we concluded that the coupled reaction involves proton transfer along the lipid membrane^26, 27^. However, in the earlier study we could not discriminate between lipid-dependent changes in the lateral proton-transfer rate or changes in protein-protein interactions. Here, we investigated direct interactions between the two membrane-bound proteins and found that these interactions correlated with the coupled ATP-synthesis activity.

We used two different combinations of protein-attached fluorophores; cyt. *bo*_3_ was labeled with either ATTO 647N or Abberior STAR 635 while the ATP-synthase was always labeled with ATTO 594. The ATTO 647N dye is hydrophobic while Abberior STAR 635 and ATTO 594 are more hydrophilic^31–33^. These properties were used to address questions related to dye-mediated interactions between the protein complexes.

We note that upon labeling of one population of cyt. *bo*_3_ with ATTO 647N (Fig. 4A), the diffusion was a factor of ~2 slower than when using Abberior STAR 635 (compare to data in Fig. 4C). This observation is consistent with earlier reports indicating that the hydrophobicity/polarity of the dye could have an effect on the diffusion coefficient^32^. The presence of ATTO 647N also affected the apparent diffusion time of the cyt. *bo*_3_ population that was labeled with ATTO 594 in the same sample (Fig. 4A). This observation indicates some degree of interaction between the two protein populations, as also observed when using FCCS (see below).

The ATP synthase displayed a factor of ~2 slower diffusion than cyt. *bo*_3_ (which cannot be attributed to the dye). This difference in diffusion constants between cyt. *bo*_3_ and the ATP-synthase, in combination with the dye-dependent differences in interactions between the proteins, was used here as a tool to investigate interactions between the two proteins. The origin of the slower ATP-synthase diffusion compared to that of cyt. *bo*_3_ is outside the scope of this work (it will be followed up in a future study) and we only discuss the effect briefly. Results from a recent study indicate that the lateral diffusion of a membrane protein is primarily determined by protein-induced deformation of the membrane, rather than the size of the protein^34, 35^. Thus, even though the ATP-synthase is larger than cyt. *bo*_3_ and has a significantly larger domain penetrating into solution outside of the membrane, the observed effect is more likely to originate from the fact that the ATP-synthase bends the membrane. The mitochondrial ATP-synthase dimer induces a curvature of ~90° in the membrane, while the monomeric form is thought to induce a bending of ~45°^36, 37^. In bacteria, there are so far no reports of a dimeric form of ATP-synthase, but because also the monomeric form is expected to induce membrane bending, the slower diffusion could be explained in terms of bending. It is also interesting to note that under conditions where cyt. *bo*_3_ and the ATP-synthase do interact (see e.g. Fig. 4B, left-hand side) the diffusion coefficient of the cyt. *bo*_3_-ATP-synthase complex is about the same as that of ATP-synthase alone, which suggests that the larger size of the cyt. *bo*_3_-ATP-synthase multi-protein complex does not slow the diffusion.

The FCCS data showed that in all samples there was a cross-correlation amplitude larger than zero, indicating some degree of protein-protein interactions. No cross-correlation was observed in an experiment using labeled DPPE lipids (i.e. ATTO 647N and ATTO 594), incorporated in GUVs at similar concentrations (data not shwon), which indicates that the fluorophores alone do not bind each other and that the contribution from spectral cross-talk between the detection channels to the FCCS amplitudes can be neglected. We normalized the data to the maximum theoretical cross-correlation amplitude, which was set to unity (Figs 3C,F and 5, see also Materials and Methods section for details). However, there are several reasons why the maximum observed amplitude may be lower than the theoretical value. For example, protein-protein interactions are likely to depend on the relative orientation of the interacting proteins in the membrane such that only proteins in the correct relative orientation would give rise to a cross correlation. Furthermore, we used a lower concentration of one of the fluorophores (i.e. ATTO 594) to eliminate spectral cross talk in the FCCS, but this also resulted in a lower maximal cross-correlation amplitude in the experiment. Thus, the absolute fraction of proteins that interact could not be determined accurately. Nevertheless, a comparison of amplitudes between samples is relevant, which is the basis of the conclusions drawn here.

The cross-correlation analysis shows that the cyt. *bo*_3_-ATP-synthase interaction in a DOPC membrane was three-fold larger than the cyt. *bo*_3_-cyt. *bo*_3_ cross correlation (Fig. 5A, blue bars). This difference was not observed upon addition of 5% DOPG to the DOPC membrane (Fig. 5A, green bars), which indicates that introduction of DOPG into the DOPC membrane resulted in weakened cyt. *bo*_3_-ATP-synthase interactions. This conclusion is also supported by the data in Fig. 4 showing that the diffusion coefficient of cyt. *bo*_3_ decreased significantly upon introduction of ATP synthase into the membrane.

When using Abberior STAR 635 as a label for cyt. *bo*_3_ in DOPC membranes (Fig. 5B, left), there was a smaller difference in cross correlation between samples containing cyt. *bo*_3_-cyt. *bo*_3_ and those containing cyt. *bo*_3_-ATP synthase than when using ATTO 647N (Fig. 5A, left), respectively. Moreover, the effect of introducing negatively charged lipids (DOPG) was also less pronounced when cyt. *bo*_3_ was labeled with Abberior STAR 635 (Fig. 5B, right). Both the cross-correlation data (Fig. 5) and the diffusion data (Fig. 4) indicate that the interactions between cyt. *bo*_3_ and the ATP-synthase were more pronounced with the ATTO 594/ATTO 647N pair of dyes than with the ATTO 594/Abberior STAR 635 pair of dyes. In other words, the interactions between cyt. *bo*_3_ and ATP-synthase could be promoted when the hydrophobic ATTO 647N dye was used. Measurements of the ATP synthesis activity (Fig. 6) show that the dye-promoted interactions result in an increase in the coupled activity. The data show that the activity was three times larger when using the ATTO 594/ATTO 647N pair of labels than when using the ATTO 594/Abberior STAR 635 pair (Fig. 6B). Furthermore, when using the ATTO 594/ATTO 647N pair of labels the activity was five times higher than for that measured with unlabeled proteins. Importantly, the lipid dependence was significantly less pronounced in the ATTO 674N/ATTO 594 sample than with unlabeled proteins (Fig. 6C). In other words, the interactions between cyt. *bo*_3_ and the ATP-synthase, promoted by the dyes, were resistant towards introduction of DOPG into the membrane.

It should be noted that in the GUVs, used for the FCS and FCCS experiments, the protein density in the membrane was ~10 times lower (~30 proteins/μm^2^) than in the functional studies using 100-nm vesicles (~320 proteins/μm^2^). Therefore, we can only do qualitative comparisons of the data from these two sets of experiments. Furthermore, we note that in our earlier studies we found that a decrease in the protein density from ~320 proteins/μm^2^ to ~80 proteins/μm^2^ resulted in significant loss of the lipid dependence of the activity. Consequently, we propose that the reason why we observe interactions in the GUVs (FCS experiments) even though the protein density is lower than ~80 proteins/μm^2^ is the ability of the ATTO 647N dye to promote interactions between the cyt. *bo*_3_ and the ATP-synthase, i.e. we would not expect to see interactions between cyt. *bo*_3_ and ATP-synthase in the GUVs without the use of the dyes. In other words, as already mentioned above, the ATTO dye itself is a tool to promote and modulate protein-protein interactions, which allowed us to study effects of lipid composition on the coupled activity also with the lower protein density in the GUVs (than in the large unilamellar vesicles). These dyes most likely promote interactions between any membrane proteins, depending on the site of labeling and number of labels. Therefore, the effect should be considered when using these dyes in other studies aimed at investigating protein-protein interactions.

**Figure 7:**
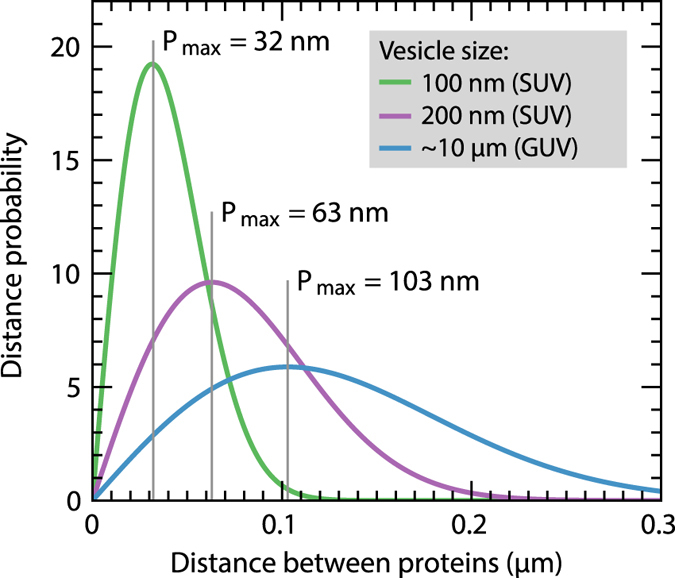
Calculated distance distribution of proteins in vesicles. Distance-probability distribution between cyt. *bo*_3_ and ATP-synthase for the protein concentration in the ~10 μm GUVs (30 proteins/μm^2^) as well as for the 200 nm (80 proteins/μm^2^) and 100 nm (320 proteins/μm^2^) large unilamellar vesicles. The distance of highest probability (*P*_max_) is indicated in the figure. The probability distribution was obtained by calculating (see e.g. ref. 52) the probability of finding the nearest-neighbor to a particle in two dimensions for distances 0–300 nm.

The distribution of the distances between cyt. *bo*_3_ and ATP-synthase range up to ~80 nm for the 100-nm vesicles (Fig. 7), i.e. under conditions where we did observe a lipid dependence of the coupled activity the maximum distance between the membrane-bound proteins was ~80 nm. This experimentally estimated distance of ~80 nm is consistent with the surface area of ~10^4^ nm^2^ (radius ≅ 60 nm) at which protons ejected from a single proton pump spread before being released to bulk solution^38^. The value is also in agreement with data from earlier experimental and theoretical studies^5, 22, 25, 39–41^.

We may also ask whether or not the mechanism has a functional role in the living cell. In mitochondria, the respiratory chain (i.e. the proton pumps and transporters) and the ATP-synthases are physically separated in the cristae (reviewed in ref. 42). The size of the cristae is of the same order as the maximum average distance between cyt. *bo*_3_ and ATP-synthase in the 100-nm vesicles, i.e. ~80 nm (Fig. 7). Consequently, it is possible that protons that are pumped or translocated by components of the respiratory chain are transferred along the membrane surface to the ATP-synthase^42, 43^.

A system in which proton transfer along membrane surfaces is particularly important to ATP synthesis is in alkaliphilic bacteria as outlined in the introduction section. In the native system where membrane proteins diffuse more freely, direct interactions could be promoted by regulatory small, membrane-bound proteins^44–47^, which would have a similar role to the hydrophobic probe used here. The findings from this study point to a mechanism by which the cell could exercise such a regulation of the energy-conversion rate (rate of ATP-synthesis) by altering the distance between the two proteins.

Unrelated to the present study, but potentially interesting in studies where protein-protein interactions are investigated, is the effect of the hydrophobic dyes to promote protein-protein interactions. In other words, care should be exercised when interpreting results from studies of interactions when these fluorophores are used to detect the proteins of interest.

## Summary

As outlined above, results from earlier studies indicate that the membrane surface is involved in proton transfer between membrane proteins. The data presented in this work show that in GUVs (where the proteins are diluted in the plane of the membrane by the large membrane surface area) direct interactions between cyt. *bo*_3_ and ATP synthase were promoted by binding of hydrophobic fluorescent dyes (which were also used to study the interactions). Binding of these dyes also resulted in an increase in the ATP-synthesis rate. Both the physical protein-protein interactions and the coupled activity dropped upon introduction of the negatively charged DOPG into the DOPC vesicles. In other words, we found a link between average lateral distance and the coupled activity. Consequently, the earlier observed lipid dependence of the coupled activity can be explained by changes in the average protein-protein distance. We also found that lateral proton transfer along the membrane occurs over distances ranging up to ~80 nm.

## Materials and Methods

### Protein expression and purification

F_1_F_o_ ATP synthase was expressed from plasmid pBWU13-βHis in *E*. *coli* strain DK8 and purified as described^48^. The quinol-type oxidase cyt. *bo*_3_ was expressed from plasmid pETcyo in *E*. *coli* strain C43 and purified as described (ref. 49, see also ref. 50).

### Protein labeling

The proteins were labeled with either of the three thiol-reactive fluorophores ATTO 594, ATTO 647N (ATTO TEC GmbH), or Abberior STAR 635 (Abberior GmbH). ATP synthase was labeled with ATTO 594 by adding a 3-fold molar excess of the dye and the sample was incubated while gently shaking for 1.5 h at room temperature. Cyt. *bo*_3_ was labeled with ATTO 647N by adding a 1.3-fold molar excess dye or with Abberior STAR 635 by adding a 5-fold molar excess of dye in the presence of 1/20 volume of NaHCO_3_ (pH 9.0), and incubated as described above. Unbound dye was removed using a pre-packet gel filtration column (PD-10, GE Healthcare) equilibrated with a 10 mM phosphate buffer (pH 7.4) supplemented with 100 mM sucrose, 10 mM KCl and 1 mM DDM. The proteins were stored in the same buffer at −70 °C until use.

The labels were attached to cysteine residues using thiol-reactive dyes. The degree of labeling was estimated using both absorbance spectroscopy and so called fluorescence antibunching^51^, where the number of independently emitting fluorophores within a molecule is determined; cyt. *bo*_3_ was on average labeled with four fluorophores per protein whereas ATP synthase was labeled with three. From a simple solvent accessibility analysis, using a probe radius of 1.4 Å, six of the seven cysteine residues found in the structure of cyt. *bo*_3_ (PDB id: 1FFT) are surface exposed. The F_1_ part of the ATP synthase has 17 cysteines (PDB id: 1OAA), of which three are available at the surface.

### Luminescence assay

Vesicles were prepared from 1,2-dioleoyl-sn-glycero-3-phosphocholine (DOPC) and 1,2-dioleoyl-sn-glycero-3-phospho-(1′-rac-glycerol) (DOPG) purchased from Avanti Polar Lipids Inc. The lipids were stored in chloroform at −20 °C until use. The lipid stock solutions were mixed at specific ratios (see Figure legends) and the chloroform was evaporated under nitrogen followed by vacuum evaporation. The lipid mixture was re-suspended at a 5 mg/ml lipid concentration in a buffer containing 10 mM HEPES pH 7.4, 2.5 mM MgCl_2_, 50 g/l sucrose and subjected to 6 freeze-thaw cycles (one minute in liquid nitrogen, then at 30 °C until thawed, followed by 30 s vortexing). Finally, vesicles were formed by extrusion (>20 times) of the mixture through 100 nm Nuclepore membranes (Whatman Ltd). Enzymes were reconstituted as previously described^26^. Briefly, a solution of 0.37 µM ATP synthase (unlabeled or labeled with ATTO 594) and 0.37 µM cyt. *bo*_3_ (unlabeled or labeled with either ATTO 647 N or Abberior STAR 635) was mixed with 0.07 µM vesicles in the presence of 0.4% sodium cholate. Samples were incubated on ice for 30 minutes. The detergent was removed using a pre-packed gel filtration column (PD-10, GE healthcare). Coupled cyt. *bo*_3_-ATP synthase activity was measured as described earlier^26, 27^. Briefly, to 460 μl of measuring buffer (20 mM Tris-PO_4_ pH 7.5, 2.5 mM MgCl_2_, 2 mM DTT, 80 μM ADP) we added 20 μl of a 10 mg/ml luciferase/luciferin solution (CLSII, Roche) and 20 μl of the proteoliposome solution. A baseline was recorded. An addition of 2 μl ATP (2.5 μM) was made and a new baseline was recorded for calibration purposes. The reaction was started by addition of 1 μl ubiquinol Q_1_ (10 mM) and ATP synthesis was recorded for 3 × 30 s.

### Preparation of giant unilamellar vesicles

The GUVs were prepared by electroformation using a modified version of the method described in ref. 28. The stock solutions of lipids (Avanti Polar Lipids), 1,2-dioleoyl-sn-glycero-3-phosphocholine (DOPC) and 1,2-dioleoyl-sn-glycero-3-phospho-(1′-rac-glycerol) (DOPG), were mixed at different ratios to a final concentration of 1 mM in chloroform. In all samples 1% (0.01 mM) 1,2-dioleoyl-sn-glycero-3-phosphoethanolamine-N-(biotinyl) (DPPE-biotinyl) was supplemented to the lipid mixture. The lipid mixtures were spread on glass plates (25 μl/plate) coated with gold. The chloroform was evaporated in open air and the lipid films was then dried extensively under vacuum. A chamber filled with a 100 mM sucrose solution was created between two plates using an O-ring coated with vacuum grease (Dow Corning). The space between the plates was 1.5 mm and the chamber volume approximately 500 μl, yielding a final lipid concentration after electroformation of maximally 0.1 mM. Formation of the GUVs was done by applying an electric field across the two glass plates using a pulse generator. The electroformation was carried out at room temperature by stepwise increasing the voltage from 100 mV (RMS at 10 Hz frequency) to 1.1 V and then keeping the voltage constant for 1.5 h. This procedure was followed in time by a 30 min detaching phase at 1.3 V and 4 Hz frequency, in order to increase the yield of GUVs in solution.

### Protein reconstitution

The two proteins, cyt. *bo*_3_ and ATP synthase, were co-reconstituted into the GUVs using a mild detergent treatment with DDM (or Na-Cholate), using a protocol modified from ref. 28. First, the protein stock solutions with 1 mM DDM (or 46 mM Na-Cholate) were mixed with 20 μl GUV-solution to a final concentration of 0.05–0.25 μM protein and 0.05 mM DDM (2.3 mM Na-Cholate) and incubated at room temperature for 30 min. Then, the proteo-GUVs were diluted 20 times in a 10 mM HEPES buffer (pH 7.4) containing 100 mM glucose and 10 mM NaCl, transferred to a LabTek microscope chamber coated with streptavidin (to immobilize the GUVs by the interaction with DPPE-biotinyl) and further incubated for 2 h. The dilution gave a final detergent concentration of 2.5 μM DDM (0.12 mM Na-Cholate) in the sample. The protein concentration in the GUVs was estimated using FCS and was found to be ~30 proteins/μm^2^ (which corresponds to ~9400 proteins in a 10 μm diameter GUV).

### FCS and FCCS measurements and instrumentation

FCS measurements were performed on a confocal setup, built on an instrument from Abberior Instruments (Göttingen, Germany), including a stand from Olympus (IX83) and a four-mirror beam scanner (Quad scanner, Abberior Instruments). Two fiber-coupled, pulsed (20 MHz) diode lasers emitting at 637 nm (LDH-D-C, PicoQuant AG, Berlin) and 594 nm (Abberior Instruments) were used for excitation (alternating mode, with the excitation pulses of the two lasers out of phase with each other to minimize cross-talk). The two laser beams were overlapped and focused by a water immersion objective (Olympus, UPLSAPO 60XO, NA 1,2). The fluorescence was collected through the same objective, separated from the excitation path via a dichroic mirror, passed through a motorized confocal pinhole (MPH16, ThorLabs, set at 50 µm diameter) in the image plane, split by a dichroic mirror and then detected by two single photon counting detectors (Excelitas Technologies, SPCM-AQRH-13), equipped with separate emission filters (FF01–615/20 and FF02-685/40-25, Semrock). The two detectors were connected to two single photon counting cards respectively (SPC 150, Becker and Hickl, Berlin, Germany) enabling FCS recordings as well as lifetime measurements. Laser timing/triggering and detector gating was controlled via a FPGA-card and by the Imspector software (Abberior Instruments).

The auto correlation functions for FCS and FCCS (G(*τ*) and G_GR_(*τ*), respectively) *τ* were calculated as described in ref. 30. The normalized cross-correlation amplitudes, G_GR_(0), were calculated as follows (but see also ref. 30):

3 $${G}_{GR}(0)=\frac{{N}_{GR}}{({N}_{G}+{N}_{GR})({N}_{R}+{N}_{GR})}$$

where *N*_GR_ is the average number of clusters within the detection area and *N*_G_, *N*_R_ is the average number of green (here ATP synthase labeled with ATTO 594) and red (here cyt. *bo*_3_ labeled with ATTO 647 N or Abberior STAR 635) labeled free diffusing, unbound, protein in the detection area. The total number of diffusing specimens within the diffraction area (including clusters and unbound proteins) are *N*_GR_/*N*_tot_, where *N*_tot_ = *N*_G_ + *N*_R_ + *N*_GR_. This gives a number between 0 and 1 where 0 means that there are no cluster formations (assuming no spectral cross-talk between the detection channel) and 1 means that all green and red labeled proteins interact to form clusters. The auto correlation amplitudes for each species can be expressed as,

4 $${G}_{G}(0)=\,\frac{1}{{N}_{G}+{N}_{GR}}$$

5 $${G}_{R}(0)=\,\frac{1}{{N}_{R}+{N}_{GR}}$$

Combining Eqs (4) and (5) with Eq. (3) gives the desired result for

6 $${{\rm{N}}}_{{\rm{GR}}}/{{\rm{N}}}_{{\rm{tot}}}=\frac{{{\rm{N}}}_{{\rm{GR}}}}{{{\rm{N}}}_{{\rm{G}}}+{{\rm{N}}}_{{\rm{R}}}+{{\rm{N}}}_{{\rm{GR}}}}=\,\frac{{{\rm{G}}}_{{\rm{GR}}}(0)}{{{\rm{G}}}_{{\rm{G}}}(0)+{{\rm{G}}}_{{\rm{R}}}(0)-{{\rm{G}}}_{{\rm{GR}}}(0)}$$

from which it is possible to estimate the fraction of clusters from the experimentally obtained amplitudes of the cross-correlation and auto correlation curves.
